# Tumor Microenvironment Specifically Regulated Nano Chemoamplifier for Chemosensitization and Activation of Anti-Tumor Immune Response by Coordinating Intracellular Magnesium Overload

**DOI:** 10.3390/pharmaceutics17081034

**Published:** 2025-08-09

**Authors:** Chao Liu, Gaofei Huang, Lu Zhu, Shasha Li, Kun Yang, Nuernisha Alifu, Yingni Duan

**Affiliations:** 1 School of Medical Engineering and Technology, Xinjiang Medical University, Urumqi 830017, China; cliu20@sohu.com (C.L.);; 2 Institute of Medical Engineering Interdisciplinary Research, Xinjiang Medical University, Urumqi 830017, China; 3 College of Basic Medicine, Xinjiang Medical University, Urumqi 830017, China; 4 College of Pharmacy, Xinjiang Medical University, Urumqi 830017, China; 5 College of Life Science and Technology, Huazhong University of Science and Technology, Wuhan 430074, China

**Keywords:** chemotherapy, magnesium ions, synergy therapy, immune activation, tumor treatment

## Abstract

**Background and objectives:** Chemotherapy is an established treatment modality for breast cancer; however, it is impaired by issues such as highly refractory chemoresistance and significant side effects. Magnesium ions (Mg^2+^), inorganic metal ions with anti-tumor bioactivity, sensitize cancer cells to chemotherapy by depressing P-glycoprotein (P-gp) expression. Moreover, Mg^2+^ functions as an immunoadjuvant to potentiate anti-tumor immune responses, while excessive Mg^2+^ can induce marked tumor cell apoptosis. **Methods:** To enable Mg^2+^ to serve as a chemotherapeutic adjuvant for enhanced treatment efficacy, a Trojan horse-like chemoamplifier, denoted as MMSN@Dox, endowed with tumor microenvironment (TME) responsiveness and capable of achieving chemotherapy sensitization and anti-tumor immune activation, was constructed to enhance the efficacy of breast cancer treatment. Leveraging Mg^2+^-enabled TME-responsive degradability of the chemoamplifier, density functional theory (DFT) simulations were conducted to elucidate carrier structural dynamics. **Results:** Under stimulation of TME, the chemoamplifier decomposes, accompanied by a substantial release of chemotherapeutic agents and metal ions. Excessive Mg^2+^ induces significant tumor cell apoptosis by triggering mitochondrial dysfunction and generating reactive oxygen species (ROS), and reinforces chemotherapy sensitivity by depressing P-gp expression. Furthermore, MMSN@Dox weakens the stemness of tumor cells, further enhancing chemotherapy. The remarkable tumor-killing capability of chemoamplifier MMSN@Dox led to a remarkable immunogenic cell death (ICD) effect. Combined with the regulatory function of Mg^2+^ on T cells, it ultimately activates anti-tumor immune responses and achieves exceptional anti-tumor performance in both in vitro and in vivo models. **Conclusions:** This approach, leveraging Mg^2+^ to enhance chemotherapy efficacy, establishes a new paradigm for overcoming chemotherapy resistance and offers a novel strategic avenue for advancing nanomedicine in breast cancer treatment.

## 1. Introduction

Breast cancer, a highly malignant form of cancer, is the most commonly diagnosed cancer among women and has surpassed lung cancer as the leading cause of cancer-related mortality, with its incidence showing an increasing trend year by year [1,2]. Chemotherapy, a conventional treatment modality for breast cancer, suppresses tumor cell proliferation by inducing DNA damage and significantly prolongs patient survival. Anthracycline drugs like doxorubicin (Dox) have been widely utilized in breast cancer treatment [3,4]. Despite the widespread use of chemotherapeutic agents in breast cancer treatment, severe issues such as multidrug resistance hinder their further application in clinical settings. Among the various factors contributing to chemoresistance, multidrug resistance-related proteins, such as P-gp and cancer stem cells (CSCs) are significant contributors to chemotherapy refractoriness [5,6,7]. Moreover, although emerging immunotherapy in recent years can strengthen chemotherapy outcomes, the vast majority of tumors resistant to Dox exhibit a cold immune phenotype, which further exacerbates treatment challenges and accelerates disease progression. Therefore, it is imperative to develop therapeutic modalities capable of inducing chemosensitization while simultaneously activating anti-tumor immune responses for breast cancer treatment.

In recent years, metal ions have garnered increasing attention in tumor treatment due to their therapeutic efficacy and the rapid development of metalloimmunology. For instance, Fe^2+^ and Cu^2+^ can specifically induce tumor-associated ferroptosis and cuproptosis, respectively, while Mn^2+^ can trigger ferroptosis in tumors and activate the cGAS-STING signaling pathway [8,9,10]. These have spurred researchers to develop a series of metal-ion-based cancer therapeutic platforms with remarkable anti-tumor efficacy. Although Fe^2+^ and Cu^2+^ exhibit remarkable anti-tumor efficacy, they can readily induce toxic side effects in normal tissues. In contrast, the therapeutic potential of essential physiological metal ions has not been accorded the same level of significance as high-profile metal ions like Mn^2+^. As researchers have gained a deeper understanding of metalloimmunology and the roles played by metal ions in tumorigenesis and progression, essential metal ions represented by Ca^2+^ and Mg^2+^ have garnered increasing attention from the scientific community [11,12,13]. Compared to the heightened attention Ca^2+^ has received for its role in regulating mitochondrial function in tumor cells and thereby influencing tumorigenesis and progression, the role of Mg^2+^ in tumor treatment has been somewhat dimmer. Nevertheless, recent studies have revealed that excessive Mg^2+^ in breast cancer cells leads to elevated levels of ROS and induces apoptosis [14,15,16]. Notably, breast cancer patients exhibit lower plasma Mg^2+^ concentrations than healthy individuals [17], indicating that elevating Mg^2+^ concentrations in patients has significant clinical implications. Moreover, Mg^2+^ promotes tumor cell apoptosis and inhibits tumor proliferation and invasion by influencing mitochondrial function and disrupting tumor cell metabolic pathways [18,19,20,21]. More importantly, elevating intracellular Mg^2+^ concentration in tumor cells can depress the expression of P-gp, thereby enabling Mg^2+^ to diminish tumor multidrug resistance and enhance chemotherapy [22,23,24]. Thus, Mg^2+^ possesses tumor therapeutic functions and has the potential to sensitize tumors to chemotherapy. In contrast, Mg^2+^ also plays a significant role in anti-tumor immunity; for instance, Mg^2+^ can modulate T cell function and promote the activation of anti-tumor immunity and has been widely used as an immune adjuvant [17,25]. These characteristics endow Mg^2+^ with critical practical importance in the management of cancer. In summary, it is highly warranted to introduce Mg^2+^, a promising component, into drug delivery systems (DDSs) to achieve co-enrichment of drugs and Mg^2+^ at tumor sites. However, most magnesium alloys and magnesium-based nanoparticles are administered via implantation or local injection due to their significant degradability [26,27,28]. This often leads to the rapid degradation of the implant in vivo, resulting in the sudden release of Mg^2+^, which may damage the surrounding normal tissues [29]. Therefore, the development of a systematically administrable and TME-responsive intelligent DDS is of great significance for achieving precise Mg^2+^-based therapy.

Over the past few decades, the rapid advancement of nanotechnology has led to the development of numerous micro- and nano-sized particles as drug carriers to reinforce drug targeting and long-circulating capabilities in vivo, achieving commendable therapeutic effects [30,31,32]. In breast cancer treatment research, nanotechnology-based nanomedicines have also been extensively applied, markedly ameliorating the issues of poor efficacy and severe side effects associated with traditional chemotherapy, revitalizing chemotherapy, and offering the potential for broader clinical application. Among the plethora of nanoparticles, mesoporous silica nanoparticles (MSN) have garnered widespread attention from researchers due to their high surface area, targeting capabilities, and ease of functionalization. Therefore, MSN is suitable as a drug carrier and convenient for the construction of metal-ion-incorporated DDSs because of its ease of functionalization [33,34]. Notably, incorporating metal ions into MSN-based DDSs could harness the therapeutic efficiency of metal ions and endow DDSs with TME responsiveness [35]. In summary, MSN is suitable as a vehicle for co-delivering Mg^2+^ and chemotherapeutic agents to tumor cells, enabling Mg^2+^ mediated chemoamplification while achieving stimuli-responsive drug release.

Given the superior loading properties of MSN, the outstanding anti-tumor bioactivity of Mg^2+^, and their precise regulation of drug loading and release dynamics in MSN-based DDSs. This enables MSNs to act as Trojan horses to deliver Mg^2+^ and chemotherapeutic agents into tumor cells, thereby boosting chemotherapy and activating immune responses. Consequently, Mg^2+^-incorporated MSN (MMSN) was prepared using the sol-gel method. Through coordination interactions, the chemotherapeutic agent Dox was loaded, resulting in the chemoamplifier MMSN@Dox. To gain a deeper understanding of how Mg^2+^ incorporation improves the biodegradability of MMSN@Dox, the structural stability of MMSN was evaluated using DFT, and the DFT analysis results were consistent with our experimental findings. In subsequent in vitro anti-tumor evaluations, MMSN@Dox demonstrated excellent breast cancer chemotherapy amplification and immune activation capabilities, as well as effectively depressing tumor cell stemness. In an in vivo breast cancer model, MMSN@Dox exhibited significant therapeutic efficacy and the capacity to activate anti-tumor immune responses. This simple yet effective nano chemoamplifier provides inspiration and fresh alternatives for breast cancer treatment and the development of novel chemotherapeutic nanomedicines.

## 2. Materials and Methods

### 2.1. Preparation of MMSN and MMSN@Dox

MMSN was prepared based on a modified version of a previously published protocol [32,33]. Briefly, 80 mg of CTAB was dissolved in a mixture of ethanol and deionized water (27 mL/13 mL). After adding 0.5 mL of ammonia solution, the mixture was stirred at 30 °C for 30 min. Subsequently, 0.5 mL of TEOS was slowly added to the solution. After 30 min, 0.6 g of magnesium nitrate was added, and the reaction was allowed to proceed for an additional 6 h. Nanoparticles were collected by centrifugation, followed by repeated washing with deionized water and ethanol. To remove CTAB, the dried nanoparticles were calcined at 550 °C for 6 h in a furnace, yielding MMSN. For drug loading, 60 mg of MMSN and 6 mg of Dox were dispersed in 25 mL of PBS and stirred for 24 h. Dox-loaded nanoparticles (MMSN@Dox) were collected by centrifugation, thoroughly washed with deionized water, and freeze-dried. Drug loading (DL%) capacity and encapsulation efficiency (EE%) of MMSN@Dox were determined by measuring the ultraviolet absorption of the washing solution using a UV-vis spectrophotometer.

### 2.2. In Vitro Biodegradation and Drug Release

To evaluate the biodegradation performance of MMSN under physiological and TME conditions, equal amounts of MMSN (5 mg) were dispersed in PBS solutions with pH values of 7.4 and 5.6. The mixtures were incubated in a shaking incubator (37 °C, 150 rpm). At predetermined time points, an equal volume of the mixture was collected and analyzed using TEM and DLS to assess the nanoparticle morphology and structural integrity. Equal volumes of supernatant were collected at different time points, and the Mg^2+^ concentration was measured using ICP-OES to investigate the Mg2 + release kinetics under varying pH conditions.

Drug release involved dispersing equal amounts of MMSN@Dox in PBS to simulate normal physiological and TME. Initially, equal amounts of MMSN@Dox (2.3 mg) were dispersed in 2 mL of buffer solutions with pH values of 5.6 and 7.4 and placed in dialysis bags with a molecular weight cut-off of 3500 Da. They were subsequently transferred into 20 mL of the corresponding pH buffer solutions. The drug release system was incubated in a constant-temperature shaker (37 °C, 150 rpm). At predetermined time intervals, 2 mL of dialysate was collected and replaced with an equal volume of fresh buffer solution. The cumulative release amount of Dox was determined by the UV absorption of the dialysate at each time point and calculated according to Equation (1),

(1) $$Accumulative\;release\;amount=\frac{W_{i}}{W_{total}}\times 100\%$$

where *W_i_* represents the accumulative mass of the drug released at the specific time point, *W_total_* refers to the total drug loading in the nanoparticles.

### 2.3. In Vitro Cytotoxicity and Anti-Tumor Assessment

The cytotoxicity of various samples against 4T1 cells was evaluated using a CCK-8 assay kit (Yeasen, Shanghai, China). 4T1 cells were seeded in 96-well plates (1 × 10^4^ cells per well) and incubated overnight. Subsequently, the culture medium was replaced with fresh medium containing different samples (MSN, Dox, MMSN, and MMSN@Dox). After 24 h of co-incubation, 10 μL of CCK-8 reagent was added to each well, followed by an additional 2 h of incubation. The absorbance at 450 nm was measured using a microplate reader, and cell viability was calculated accordingly. To further assess the anti-tumor performance of the various samples, 4T1 cells were seeded into 6-well plates (3 × 10^5^ cells per well) and incubated overnight. The medium was then replaced with a fresh medium containing Dox (5 μg/mL), MMSN (95 μg/mL), or MMSN@Dox (100 μg/mL). Following different incubation periods (12 h and 24 h), the medium was discarded, and the cells were detached using trypsin without EDTA, harvested, and stained with Annexin V-FITC and 7-AAD for FCM analysis. Calcein-AM/PI live/dead staining kit was used to validate the in vitro anti-tumor efficacy. Following the same drug treatment protocol as in the apoptosis assay, cells were collected, stained with Calcein-AM/PI, incubated in culture medium at 37 °C for 20 min, and observed under an inverted fluorescence microscope to record live/dead cell ratios.

### 2.4. Mitochondrial Membrane Potential and Intracellular ROS Evaluation

The changes in the mitochondrial membrane potential (MMP) of tumor cells treated with MMSN were assessed using the JC-1 MMP assay kit (Beyotime, Shanghai, China) and analyzed by FCM. 4T1 cells were seeded into 6-well plates at a density of 3 × 10^5^ cells per well and cultured overnight. The cells were then co-cultured in a medium containing 95 μg/mL MMSN or MSN for 12 h. After repeated washing with PBS, the cells were harvested and washed with pre-cooled PBS. The collected cells were stained with JC-1 reagent according to the manufacturer’s protocol and subjected to FCM analysis.

To evaluate intracellular ROS levels in tumor cells after MMSN treatment, cells treated with different formulations for different periods (12 h, 24 h, and 36 h) were collected and incubated with DCFH-DA solution for 20 min, and then washed three times with PBS to remove the unbound DCFH-DA. Intracellular ROS levels were then quantified using FCM.

### 2.5. In Vitro Evaluation of ICD and DCs Maturation

DAMPs, represented by CRT and HMGB1, are critical hallmarks of ICD. To evaluate the ICD effects on tumor cells treated with different formulations, 4T1 cells seeded in 6-well plates were co-cultured with medium containing Dox, MMS, or MMSN@Dox. After 12 h, the culture supernatant was collected, and extracellular HMGB1 levels were quantified using an HMGB1 enzyme-linked immunosorbent assay (ELISA) kit. To detect cell surface CRT expression, 4T1 cells were treated under the same conditions for 12 h, harvested, and washed. The collected cells were then incubated with FITC anti-calreticulin antibody at room temperature for 30 min. Unbound antibodies were removed by washing with PBS, and CRT expression was analyzed using FCM.

The mouse bone marrow-derived dendritic cell line DC2.4 was used to evaluate DCs maturation. First, 4T1 cells were seeded into 6-well plates at a density of 3 × 10^5^ cells per well and incubated overnight. The cells were then co-cultured with Dox, MMSN, or equivalent-dose MMSN@Dox for 12 h. Subsequently, culture medium supernatants were added to immature DC2.4 cells and co-cultured for an additional 12 h. The treated DC2.4 cells were harvested, stained with BV610 anti-mouse CD80 antibody and APC anti-mouse CD86 antibody for 30 min, and washed twice with PBS. The proportion of mature DCs was analyzed using FCM.

### 2.6. In Vitro Evaluation of CSCs

To investigate the stemness of 4T1 cells after different treatments, 4T1 cells were first seeded into 6-well plates (3 × 10^5^ cells per well) and incubated overnight. The cells were then treated with different formulations for 12 h. Following treatment, the cells were harvested and stained with PerCP/Cy5.5 anti-mouse CD44 antibody and FITC anti-mouse CD24 antibody. After thorough washing with PBS, the cells were analyzed by FCM.

### 2.7. In Vivo Anti-Tumor Evaluation

Seven-week-old Balb/c mice were used to establish a 4T1 subcutaneous tumor model. A total of 1.5 × 10^6^ 4T1 cells were subcutaneously inoculated into mice. When the tumor volume reached about 150 mm^3^, tumor-bearing mice were randomly divided into four groups: Normal saline, Dox, MMSN, and MMSN@Dox. Therapeutic agents were administered via tail vein intravenous injection (i.v.) on days 1, 3, and 5, with a Dox dosage of 3 mg/kg. Tumor size was recorded every two days using calipers, and tumor volume was calculated according to Equation (2),

(2) $$Volume=\frac{\left(L\times W^{2}\right)}{2}$$

where L and *W* represent the length and width of tumor, respectively. Body weight was recorded every two days. After 16 days, tumors from each group were excised, weighed, and photographed, followed by H&E and TUNEL staining analysis.

### 2.8. In Vivo Anti-Tumor Immune Response Evaluation

To evaluate anti-tumor immune activation and T cell infiltration, tumor-bearing mice were administered therapeutic agents via i.v. on days 1, 3, and 5. On day 7, the mice were euthanized, and the tumors and tumor-draining lymph nodes (TDLNs) were harvested. TDLNs were dissociated into single-cell suspensions. The cells were stained with FITC anti-mouse CD11c, BV610 anti-mouse CD80, APC anti-mouse CD86, and PerCP/Cy5.5 anti-mouse MHC-II antibodies. FCM analysis was performed to quantify DCs’ maturation markers. At the same time, tumors were minced and digested with collagenase to generate single-cell suspensions, followed by the removal of red blood cells using red blood cell lysis buffer and subsequent washing with PBS twice. The resulting cells were stained with APC/Cy7 anti-mouse CD45, PerCP/Cy5.5 anti-mouse CD3, BV421 anti-mouse CD4, PerCP anti-mouse CD8, and FITC anti-mouse PD-1 antibodies. After washing, immune cell subsets and checkpoint marker expression were analyzed using FCM.

### 2.9. Statistical Analysis

All experimental data are expressed as the mean ± standard deviation (SD). Statistical analysis was performed using GraphPad Prism 9.5.1 software. For comparisons involving more than two groups, statistical differences were analyzed using one-way analysis of variance (ANOVA) followed by Tukey’s test. The number of replicates *n* for each experiment is indicated in the figure legend. A p-value less than 0.05 (*p* < 0.05) was considered statistically significant, and * *p* < 0.05, ** *p* < 0.01, *** *p* < 0.001, **** *p* < 0.0001.

## 3. Results

### 3.1. Preparation and Characterization of MMSN

**Figure 1:**
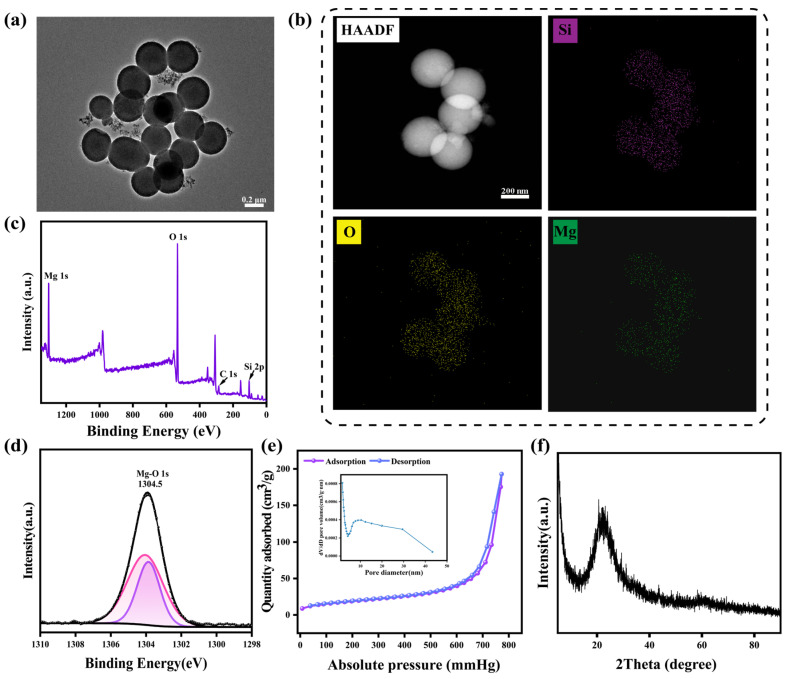
Characterization of MMSN. (**a**) TEM image of MMSN and (**b**) TEM mapping results of MMSN. (**c**) XPS pattern and (**d**) deconvoluted Mg 1s spectra of MMSN. (**e**) Nitrogen adsorption-desorption isotherms and pore size distribution of MMSN. (**f**) XRD pattern of MMSN.

MMSN was prepared using a sol-gel method. Transmission electron microscopy (TEM) revealed that the MMSN possessed a uniform spherical structure with a particle size of approximately 375 nm (Figure 1a). To validate the successful incorporation of Mg^2+^, TEM elemental mapping was employed to characterize the primary elements of the synthesized nanoparticles. As shown in Figure 1b, Mg, Si, and O elements were uniformly distributed throughout the nanoparticles, confirming the successful incorporation of Mg^2+^. Additionally, to further verify the elemental composition of the MMSN, we conducted X-ray photoelectron spectroscopy (XPS) analysis of the prepared nanoparticles. As illustrated in Figure 1c,d, the nanoparticles primarily consist of Si, O, and Mg elements, with Mg predominantly existing in the form of Mg^2+,^ and Mg-O bonds were established in the nanoparticles. This further corroborates the successful incorporation of Mg^2+^ and the successful preparation of MMSN. To accurately determine the content of Mg^2+^ in MMSN, Inductively Coupled Plasma Optical Emission Spectroscopy (ICP-OES) was utilized to quantify the Mg^2+^, and the Mg^2+^ content was found to be 14.35% (Figure S1). As a porous nanostructured material, the structure of MMSN was characterized using nitrogen adsorption-desorption analysis. Figure 1e reveals that MMSN exhibits a classic Type IV isotherm curve, indicative of its mesoporous structure. The specific surface area, pore volume, and pore diameter of MMSN were 357.01 m^2^/g, 0.27 cm^3^/g, and 9.53 nm, respectively. The excellent specific surface area and appropriate pore size facilitate the use of MMSN as a proper carrier for loading therapeutic agents. Simultaneously, XRD was used to characterize the structure of the MMSN. Consistent with the literature, MMSN exhibited only an amorphous peak near 20° (Figure 1f), indicating that MMSN possesses an amorphous structure [36].

### 3.2. TME Stimuli Responsive Drug Release

**Figure 2:**
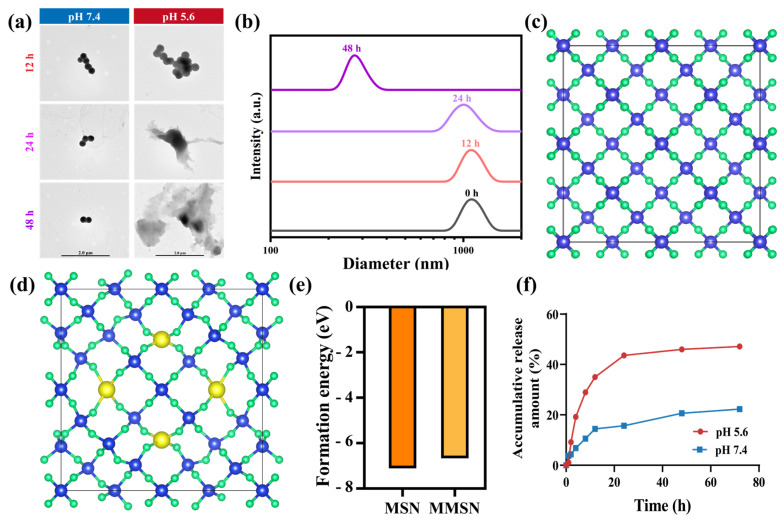
Stimuli-responsive biodegradation and drug release evaluation of MMSN. (**a**) TEM images of MMSN immersed in buffer solutions with different pH values at various time points. (**b**) Hydrodynamic diameter results of MMSN immersed in acidic buffer (pH 5.6) at different time points. Molecular structure diagrams of (**c**) MSN and (**d**) MMSN, (**e**) corresponding formation energy results of MSN and MMSN. (**f**) Dox release profile of MMSN@Dox in different buffer solutions.

MSN, which are drug carriers with outstanding drug-loading capacities, have long faced a critical barrier to clinical translation due to their poor biodegradability. Among all strategies, incorporating metal ions into MSN to form metal-oxygen bonds can significantly improve biodegradability and endow MSN with TME-responsive drug release properties. To validate the biodegradability of MMSN, the nanoparticles were immersed in buffer solutions with pH values of 5.6 and 7.4, and the particle size and morphology were monitored over time. As shown in Figure 2a, the TEM results revealed that when MMSN was incubated in acidic buffer (pH 5.6), although it retained a regular spherical structure at 12 h, its morphology became irregular by 24 h, indicating that significant biodegradation had occurred. By 48 h, the structure of MMSN became completely irregular, demonstrating its gradual degradation over time in the acidic buffer. In contrast, when the MMSN was immersed in a neutral buffer (pH 7.4), it retained its structural integrity even after 48 h, demonstrating that no observable degradation occurred. To fully validate the degradation of MMSN under acidic conditions, we measured the hydrodynamic diameter of MMSN in acidic buffer at different time points. As depicted in Figure 2b, the hydration particle size of MMSN was still close to its initial state at 12 h, and at 24 h, its particle size diminished to about 800 nm, indicating that degradation had occurred, leading to a reduction in size. By 48 h, the particle size of MMSN had further shrunk to 230 nm, indicating that the degree of degradation had further intensified, which was consistent with the results obtained from TEM. These results confirm that MMSN undergo biodegradation in acidic environments.

The improvement of biodegradability by metal ion incorporation has been extensively confirmed by researchers. However, the underlying theoretical mechanisms of this phenomenon remain largely unexplored. Herein, we employed DFT to conduct computational simulations on the improvement of MMSN degradation by Mg^2+^ incorporation. As shown in Figure 2e, compared to MSN, MMSN exhibits a significantly higher formation energy, indicating that Mg^2+^ incorporation weakens the structural stability of silica nanoparticles, thereby rendering them more susceptible to degradation in acidic conditions. The DFT calculation and simulation results align well with our experimental results, demonstrating that the excellent responsive degradation of MMSN upon Mg^2+^ incorporation arises from the increased formation energy.

Incorporation of Mg^2+^ endows MMSN with enhanced biodegradability and significantly improves their drug-loading capacity. Compared to MSN, MMSN markedly increased the encapsulation efficiency (EE%) of the chemotherapeutic drug Dox, enabling more chemotherapeutic agent loading into the carrier by coordination interaction (Figure S2). Given MMSN’s superior degradation performance in acidic environments, this allows MMSN@Dox with TME-responsive drug release. As shown in Figure 2f, although the drug release amounts in both media were comparable within the first 2 h, the release rate in the acidic medium subsequently became significantly higher than that in the neutral medium. This is attributed to the more pronounced degradability of MMSN in acidic environments, which thereby accelerates drug release. By 24 h, the cumulative drug release content in the acidic medium was 2.78-fold higher than that in the neutral medium, demonstrating a pronounced pH-responsive drug release behavior. Meanwhile, the acidic buffer also accelerated Mg^2+^ release, reaching 2.2 times that of the neutral buffer at 48 h (Figure S3). This characteristic endows MMSN@Dox with TME-responsive drug release capability, which has significant implications for mitigating toxic side effects and strengthening therapeutic efficacy.

### 3.3. In Vitro Anti-Tumor Evaluation

**Figure 3:**
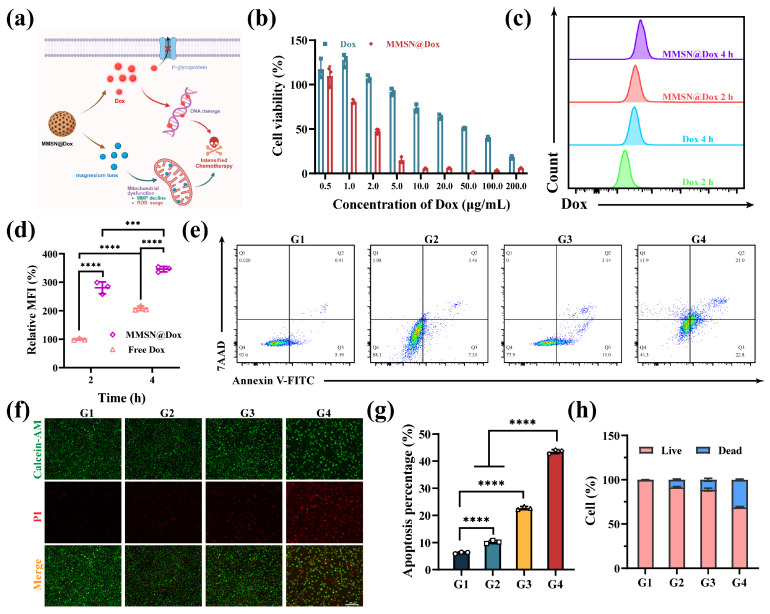
Evaluation of in vitro anti-tumor activity. (**a**) Diagram of MMSN@Dox for chemosensitization. (**b**) Cytotoxicity of 4T1 cells treated with different formulations. (**c**) FCM plots of cellular internalization and (**d**) corresponding quantitative results. (**e**) Apoptosis assay results of 4T1 cells treated with different formulations and (**g**) the quantitative statistical results. (**f**) Microscopic images of live/dead staining of 4T1 cells treated with different formulations and (**h**) the corresponding semi-quantitative statistical results. G1: Normal saline, G2: Dox, G3: MMSN, G4: MMSN@Dox. Data are shown as mean ± SD, *n* = 3, *** *p* < 0.001, **** *p* < 0.0001.

The in vitro anti-tumor efficacy of MMSN@Dox was first evaluated using the CCK-8 assay kit for cytotoxicity. After co-incubation with 4T1 cells for 24 h, MMSN@Dox exhibited more pronounced cytotoxicity than free Dox at the same concentration of Dox, with the IC50 value decreasing from 48.17 μg/mL to 2.01 μg/mL, demonstrating superior anti-tumor efficacy compared to free Dox and MMSN (Figure 3a,b, and Figure S4). A critical factor underlying the superior cytotoxicity of MMSN@Dox is that, compared to free Dox, nanoparticles facilitate greater drug internalization into cells via endocytosis. Flow cytometry (FCM) analysis of Dox fluorescence in tumor cells revealed that 4T1 cells exhibited significantly higher uptake efficiency of MMSN@Dox at all tested time points (Figure 2c,d), which was conducive to MMSN@Dox exerting a more significant anti-tumor effect. MMSN@Dox not only facilitates greater internalization of Dox by tumor cells but also exerts anti-tumor effects through excessive Mg^2+^ release due to its degradation after cellular internalization. On one hand, Mg^2+^ influences mitochondrial function and induces ROS production; on the other hand, Mg^2+^ within tumor cells impedes drug efflux, thereby strengthening therapeutic efficacy. To further validate the anti-tumor efficacy of MMSN@Dox, apoptosis assays and live/dead staining experiments were conducted to evaluate its inhibitory effects on tumor cells. In the apoptosis assay, MMSN@Dox consistently demonstrated the highest apoptosis rate, with the apoptosis proportion being 4.23-fold and 2.62-fold higher than that of free Dox at 12 and 24 h, respectively. Notably, the MMSN treatment group primarily exhibited early-stage apoptosis, which was attributed to mitochondrial dysfunction caused by excessive intracellular Mg^2+^ (Figure 3e,g, and Figure S5) [37]. Additionally, in the live/dead staining assay, which visualizes cell viability, we observed enhanced red fluorescence and diminished green fluorescence in tumor cells treated with MMSN@Dox. Quantitative analysis of the staining results also confirmed that the MMSN@Dox-treated group exhibited the highest proportion of dead cells (Figure 3f,h), which coincided with the results of the CCK-8 cytotoxicity assay and apoptosis experiments. The above experimental results fully demonstrate that MMSN@Dox possesses outstanding anti-tumor efficacy and a significant chemosensitization effect.

### 3.4. Magnesium Ions Disrupt Mitochondrial Function and Induce ROSs

**Figure 4:**
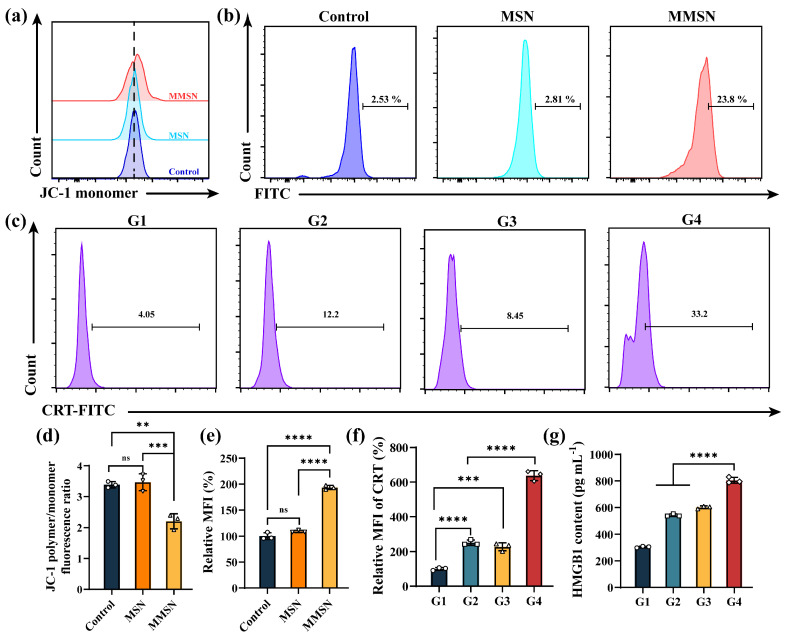
Evaluation of MMP, intracellular ROS levels, and ICD in tumor cells. (**a**) FCM plots of mitochondrial JC-1 monomers in tumor cells subjected to various treatments. (**b**) FCM profiles of Intracellular ROS levels and (**e**) corresponding quantitative statistical results. (**c**) CRT expression levels in 4T1 cells subjected to various treatments, (**f**) along with corresponding quantitative statistics. (**d**) The ratio of mitochondrial JC-1 polymer to monomer after different formulation treatments. (**g**) Extracellular HMGB1 release levels in 4T1 cells subjected to various treatments. G1: Normal saline, G2: Dox, G3: MMSN, G4: MMSN@Dox. Mean ± SD, *n* = 3, ** *p* < 0.01, *** *p* < 0.001, **** *p* < 0.0001 and ns: not significant.

The superior anti-tumor performance of MMSN@Dox is largely attributed to its ability to act like a Trojan horse, entering tumor cells and responsively releasing chemotherapeutic agents and Mg^2+^, which then induce mitochondrial dysfunction and generate ROS, ultimately triggering tumor cell apoptosis. Therefore, we evaluated the changes in the mitochondrial membrane potential (MMP) of tumor cells following different treatments. As shown in Figure 4a,d, the JC-1 monomer significantly increased in tumor cells after MMSN treatment, and the ratio of JC-1 polymer to monomer declined, indicating that mitochondrial function was disrupted. In contrast, MSN had no discernible effect on the mitochondrial function. Concurrently, the pronounced early apoptosis observed in the MMSN-treated groups during apoptosis assays also manifested mitochondrial dysfunction (Figure 3e). These collective results confirm that MMSN disrupts mitochondrial function in tumor cells. Furthermore, the detection of intracellular ROS in tumor cells revealed that MMSN induced more ROS production than MSN (Figure 4b,e), which aligns with prior literature reports [14]. Moreover, ROS levels showed a time-dependent increase under MMSN treatment, reaching approximately twice the level at 36 h compared to that at 12 h (Figure S6). These findings partially explain how MMSN@Dox achieves anti-tumor and chemotherapy-amplifying effects.

### 3.5. In Vitro ICD and Immune Activation

Although tumor cells undergo ICD and release tumor-associated antigens (TAAs) to activate anti-tumor immune responses following treatment with chemotherapeutic agents or exposure to external stimuli, robust ICD is critical for effectively triggering a potent anti-tumor immune response. To confirm whether the chemoamplifier MMSN@Dox amplifies anti-tumor immune responses while sensitizing chemotherapy, the primary markers of ICD, calreticulin (CRT), and high-mobility group box 1 (HMGB1), were measured. As shown in Figure 4c and f, the expression of CRT on tumor cell surfaces increased across all treatment groups compared to the Control group. Nevertheless, 4T1 cells treated with MMSN@Dox exhibited markedly higher CRT expression than those in the other groups, with the MMSN@Dox treatment group showing a 2.54-fold increase in CRT mean fluorescence intensity (MFI) relative to the free Dox treatment group. HMGB1 assay results indicated MMSN@Dox treatment induced 1.48 and 1.34 times greater extracellular HMGB1 release than DOX and MMSN treatments, respectively. Following treatment with MMSN@Dox, both CRT expression and extracellular HMGB1 levels were significantly increased, indicating that the chemoamplifier MMSN@Dox can effectively enhance ICD, which provides a favorable prerequisite for activating anti-tumor immune responses.

**Figure 5:**
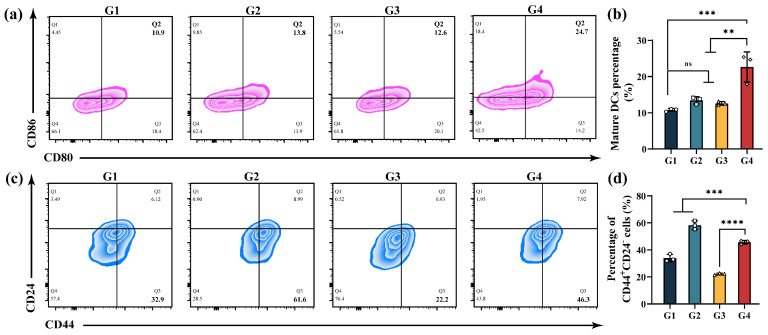
Evaluation of DC maturation and CSCs elimination. (**a**,**b**) Representative FCM plots of DC maturation after different treatments and their quantifications. (**c**,**d**) FCM profiles of sorted CD44^+^CD24^−^ cells after different formulation treatments and corresponding statistical results. G1: Normal saline, G2: Dox, G3: MMSN, G4: MMSN@Dox. Data are shown as Mean ± SD, *n* = 3, ** *p* < 0.01, *** *p* < 0.001, **** *p* < 0.0001 and ns: not significant.

During tumor progression, the immunosuppressive TME compromises the host immune system’s ability to effectively exert anti-tumor effects, resulting in a paucity of cytotoxic T cells within the tumor tissues. In tumor treatment, released DAMPs are captured by dendritic cells (DCs), a type of antigen-presenting cell, leading to DCs maturation. Matured DCs subsequently activate T cells to mount anti-tumor immune responses. Therefore, effectively promoting DC maturation and activating T cells is critical for inducing anti-tumor immune responses. To investigate the effect of MMSN@Dox treatment on DCs maturation, dendritic cells (DC2.4 cells) were co-cultured with culture supernatants from tumor cells treated with different formulations. Subsequently, FCM was used to assess the maturation status of DCs. As depicted in Figure 5a,b, compared to the free Dox and MMSN treatment groups that exhibited insignificant promotion of DCs maturation, the MMSN@Dox treatment group demonstrated a significant ability to promote DCs maturation, with the proportion of mature DCs being 2.1-fold higher than that of the Control group. This indicates that the chemoamplifier MMSN@Dox possesses a brilliant capability to activate anti-tumor immune responses.

### 3.6. In Vitro CSCs Suppression

Cancer stem cells (CSCs), a type of cell with stem cell characteristics, are key factors in the onset, progression, and drug resistance of cancer [38,39]. CSCs, in particular, exhibit remarkable resistance to conventional chemotherapy, which is an important reason for the failure of chemotherapy. To evaluate the proportion of CSCs in 4T1 cells after different treatments, FCM was used to analyze the CD44^+^CD24^−^ cell population. Experimental results revealed that a single chemotherapy treatment increased the proportion of CSCs compared to the Control group. In contrast, treatment with MMSN alone reduced the CSC population. Although the chemotherapy amplifier MMSN@Dox slightly elevated the proportion of CSCs compared to the Control group, it markedly decreased the percentage of CSCs relative to the single chemotherapy treatment group, with the proportion dropping from 58.31% in the Dox group to 45.67% in the MMSN@Dox group (Figure 5c,d). This demonstrated that the chemoamplifier MMSN@Dox could effectively suppress CSCs’ stemness, providing an alternative explanation for why MMSN enabled chemosensitization.

### 3.7. In Vivo Anti-Tumor Efficacy

**Figure 6:**
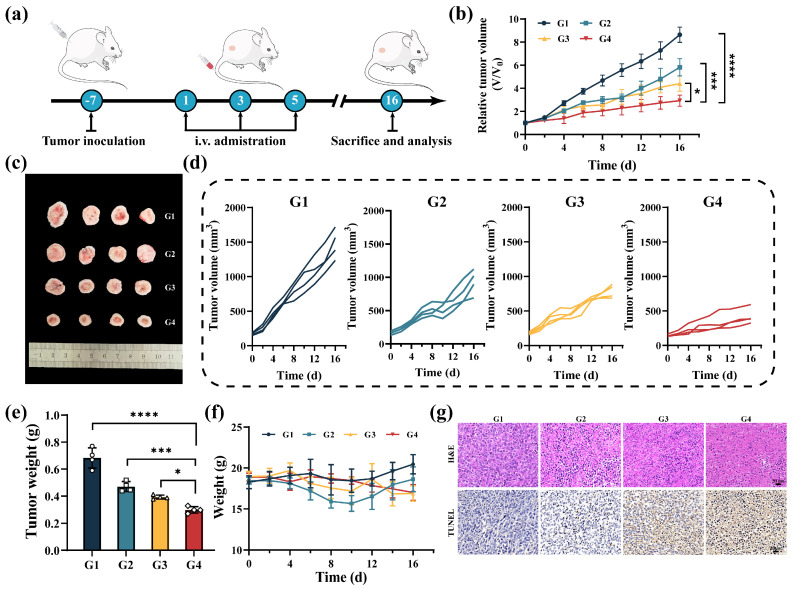
In vivo evaluation of anti-tumor efficacy on 4T1 subcutaneous tumor. (**a**) Schematic diagram of treatment protocol. (**b**) Average tumor volume growth curves in different treatment groups. (**c**) Photographs of tumors in each treatment group after 16 days. (**d**) Tumor progression curves in each treatment group. (**e**) Tumor weight in each group. (**f**) The change in body weight of mice over a period of 16 days. (**g**) H&E and TUNEL staining section images of tumors in different groups. G1: Normal saline, G2: Dox, G3: MMSN, G4: MMSN@Dox. Data are shown as Mean ± SD, *n* = 4, * *p* < 0.05, *** *p* < 0.001, **** *p* < 0.0001.

Encouraged by the remarkable anti-tumor and immunostimulatory performance of MMSN@Dox, we further evaluated anti-tumor efficacy in Balb/c mice inoculated with 4T1 tumors. When tumor volumes reached approximately 150 mm^3^, the tumor-bearing mice were randomized into four groups and administered saline, Dox, MMSN, and MMSN@Dox on days 1, 3, and 5. The experiment was terminated on day 16, and the tumors were collected for further analysis (Figure 6a). As shown in Figure 6b,d, the normal saline group exhibited rapid tumor progression, whereas tumor growth in the Dox and MMSN treatment groups was inhibited to some extent. It is noteworthy that tumor growth in the MMSN@Dox treatment group was effectively suppressed. Both the tumor photographs and tumor weights confirmed that tumors in the MMSN@Dox treatment group were significantly suppressed (Figure 6c,e), which was consistent with the tumor growth curves. In addition, analysis of tumor tissues from each group using Hematoxylin and Eosin (H&E) and Terminal Deoxynucleotidyl Transferase-mediated dUTP Nick End Labeling (TUNEL) staining revealed that the MMSN@Dox treatment group exhibited severe necrosis and apoptosis, demonstrating prominent therapeutic efficacy (Figure 6g). Tracking of mouse body weight during the treatment process revealed that, except for slight fluctuations in the Dox group, all other groups remained largely stable (Figure 6f), and H&E staining of the major organs revealed no observable pathological damage across all treatment groups (Figure S7). These results demonstrate that the chemoamplifier MMSN@Dox is safe.

### 3.8. In Vivo Anti-Tumor Immune Response

**Figure 7:**
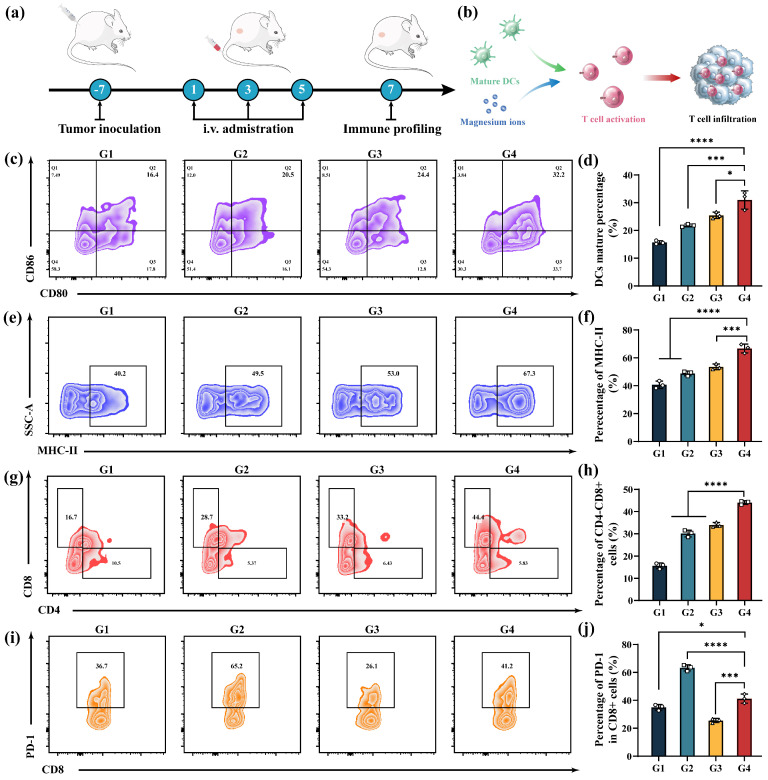
Assessment of anti-tumor immune response in vivo. (**a**) Schematic diagram of the immune evaluation experimental protocol. (**b**) Schematic illustration of anti-tumor immune response activation. (**c**,**d**) Representative FCM plots of CD80^+^CD86^+^ DCs of lymph nodes and corresponding statistical results. (**e**,**f**) FCM profiles and corresponding statistical analysis of MHC-II surface expression in DCs. (**g**,**h**) The proportion of cytotoxic T cells in tumor tissue and the corresponding statistical analysis. (**i**,**j**) FCM plots and quantification of PD-1 expression levels on cytotoxic T cells. G1: Normal saline, G2: Dox, G3: MMSN, G4: MMSN@Dox. Data are shown as Mean ± SD, *n* = 3, * *p* < 0.05, *** *p* < 0.001, **** *p* < 0.0001.

Given the robust immune activation performance of MMSN@Dox in vitro, we further analyzed the in vivo anti-tumor immune response post-treatment by evaluating the in vivo anti-tumor immune response in tumor-draining lymph nodes and tumor tissues (Figure 7a). Following the preparation of single-cell suspensions from the collected lymph nodes and tumor tissues, subsequent antibody staining and FCM analysis revealed that, compared to the other groups, the MMSN@Dox treatment group exhibited the highest DCs maturation rate, achieving 1.98-fold greater maturation than that of the saline group (Figure 7c,d). Additionally, MMSN@Dox treatment significantly upregulated MHC-II expression on dendritic cells, demonstrating a 1.56-fold increase compared to that in the saline group (Figure 7e,f). Mature DCs subsequently induce the activation and increased infiltration of T cells to exert anti-tumor immunity. As shown in Figure 7g,h, the proportion of tumor-infiltrating cytotoxic T cells (CD8^+^CD4^−^) in the MMSN@Dox treatment group significantly increased compared to that in the saline and other treatment groups, demonstrating a 2.74-fold and 1.46-fold elevation relative to the saline and Dox groups, respectively. In addition to analyzing tumor-infiltrating T cells, we examined PD-1 expression in T cells. The results revealed significantly upregulated PD-1 expression levels in the Dox treatment group (Figure 7i,j), consistent with previous reports [40,41]. Notably, heightened PD-1 expression is associated with chemotherapy resistance and also serves as a marker of T cell exhaustion, which impairs the anti-tumor effects of both chemotherapy and immunotherapy [42,43]. However, the MMSN treatment group showed a significant depression in T cell PD-1 expression. For MMSN@Dox, although the PD-1 expression level was higher than that of the MMSN group, it remained lower than that of the Dox group, being merely 65% of that of the Dox group. Meanwhile, the depression of PD-1 expression can hinder immune escape and also alleviate T cell exhaustion. Collectively, these findings demonstrate that the chemoamplifier MMSN@Dox could effectively activate anti-tumor immune responses and downregulate PD-1 expression to some extent, thereby highlighting its potential for anti-tumor immune activation and synergistic therapy with immune checkpoint inhibitors.

## 4. Conclusions

In this scenario, to strengthen the efficacy of breast cancer chemotherapy, we proposed a therapeutic strategy integrating chemosensitization with coordinated anti-tumor immunity by leveraging the therapeutic bioactivity of Mg^2+^. To operationalize this approach, an intelligent chemoamplifier MMSN@Dox based on MSNs with exceptional biodegradability was constructed in this study. The incorporated Mg^2+^ endowed the chemoamplifier with TME-responsive properties, enabling efficient stimuli-responsive drug release within the TME. Substantial Mg^2+^ released during the degradation of the chemoamplifier induced mitochondrial dysfunction and ROS generation, while simultaneously suppressing drug tolerance by downregulating tumor cell stemness and P-gp expression. Furthermore, released Mg^2+^ can modulate T cell functionality, synergizing with chemotherapy to potentiate anti-tumor effects. Briefly, this Mg^2+^-modulated nano-delivery system, MMSN@Dox, for chemotherapy sensitization, demonstrates remarkable anti-tumor efficacy and immune-activating capabilities, thereby offering a novel paradigm for advancing multifunctional nanomedicine in breast cancer chemotherapy.

## Data Availability

Data will be made available on request.

## Supplementary Materials

The following supporting information can be downloaded at: https://www.mdpi.com/article/10.3390/pharmaceutics17081034/s1. Experimental Materials, Characterization, Cell Culture, Cell internalization, and DFT Calculation Sections. Figure S1: The Mg^2+^ content in MMSN measured by ICP-OES; Figure S2: Encapsulation efficiency of MSN and MMSN; Figure S3: The cumulative release amount of Mg^2+^ by MMSN in different buffer solutions; Figure S4: Cytotoxicity of MSN and MMSN toward 4T1 cells at different concentrations; Figure S5: Apoptosis assay of 4T1 cells after being treated in different formulations for 24 h; Figure S6: Measurement of ROS after different treatment durations; Figure S7: H&E staining images of main organs in each group. References [44,45,46] are cited in the Supplementary Materials.
