# Supplementary material for: Tumor Microenvironment Specifically Regulated Nano Chemoamplifier for Chemosensitization and Activation of Anti-Tumor Immune Response by Coordinating Intracellular Magnesium Overload
Source: Pharmaceutics. 2025 Aug 9;17(8):1034. doi: 10.3390/pharmaceutics17081034 (PMC12389149; doi:10.3390/pharmaceutics17081034)
Supplement: Supplementary file 1 [file pharmaceutics-17-01034-s001.zip › pharmaceutics-3735159-supplementary.pdf]

Supplementary Materials: **Tumor Microenvironment Specifically Regulated Nano Chemoamplifier for Chemosensitization and Activation of Anti-tumor Immune Response in Breast Cancer Treatment**

Chao Liu [a, b], Gaofei Huang [a, b], Lu Zhu [c], Shasha Li [d], Kun Yang [e], Nuernisha Alifu [a, b*], Yingni Duan [a, b*]

* Corresponding authors e-mail: duanyingni123@sina.com, nens_xjmu@126.com

Materials

Cetyltrimethylammonium bromide (CTAB) was purchased from J&K Scientific Ltd. (Beijing, China). Tetraethyl orthosilicate (TEOS) was obtained from Aladdin Reagent Co., Ltd. (Shanghai, China). Ammonia solution and magnesium nitrate were provided by Sinopharm Chemical Reagent Co., Ltd. Doxorubicin hydrochloride (Dox) was acquired from Dalian Meilun Biotechnology Co., Ltd. (Dalian, China). CCK-8 assay kit, Calcein-AM Live/Dead assay kit, and apoptosis assay kit were purchased from Yeasen Biotechnology Co., Ltd. (Shanghai, China). 2',7'-Dichlorodihydrofluorescein diacetate (DCFH-DA) ROS assay kit and JC-1 mitochondrial membrane potential assay kit were provided by Beyotime Biotechnology Co., Ltd. (Shanghai, China). High Mobility Group Box 1 (HMGB1) ELISA assay kit (Cat: SEKM-0145) was acquired from Solarbio Science & Technology Co., Ltd. (Beijing, China). FITC-conjugated calreticulin antibody was procured from StressMarq Biosciences Inc. (Victoria, Canada).

Characterization and analysis

The particle size of nanoparticles was characterized by transmission electron microscopy (TEM) (HT7700, Hitachi, Japan) and dynamic light scattering (DLS) analyzer (Nano ZS90, Malvern, UK). Elemental composition was analyzed via field-emission transmission electron microscopy (FE-TEM) (Tecnai, FEI, USA) and X-ray photoelectron spectroscopy (XPS) (Escalab, ThermoFisher Scientific, USA). Specific surface area and pore volume were determined by nitrogen adsorption-desorption isotherms (ASAP 2460, Micromeritics, USA). X-ray diffraction (XRD) patterns and ultraviolet (UV) absorption spectra were recorded by X-ray diffractometer (D8 Advance, Bruker, Germany) and UV-vis spectrophotometer (TU-1901, Puxi, China), respectively. Live/dead staining results were observed and recorded using an inverted fluorescence microscope (Ti2, Nikon, Japan). Flow cytometry analysis was performed with a flow cytometer (CytoFlex S, Beckman Coulter, USA).

Cell culture

4T1 and DC2.4 cells were cultured in Roswell Park Memorial Institute (RPMI 1640) medium and Dulbecco's modified Eagle's medium (DMEM), respectively, supplemented with 10% fetal bovine serum (FBS) and 1% penicillin-streptomycin. The cells were maintained in a humidified incubator at 37°C with 5% CO_2.

DFT calculation

All calculations were performed using the first-principles pseudopotential plane-wave method implemented in the CASTEP software package [44]. The generalized gradient approximation (GGA) with Perdew-Burke-Ernzerhof (PBE) exchange-correlation functional was employed to describe electron exchange and correlation potential [45]. Plane-wave cutoff energy was set to 400 eV. A Monkhorst-Pack k-point grid of 1×1×2 was adopted for Brillouin zone sampling, and structural optimization calculations were performed with the following convergence criteria: Total energy tolerance: 2×10^{-2} eV/atom, Maximum force tolerance: 0.05 eV/nm, Maximum stress tolerance: 0.1 GPa, Maximum displacement tolerance: 0.002 Å. To improve the accuracy of electronic structure properties, the DFT-D3 correction method proposed by Grimme was utilized to account for van der Waals interactions [46].

Cell internalization

4T1 cells were seeded into 6-well plates at a density of 3×10^5 cells per well and incubated overnight. The culture medium was then replaced with fresh medium containing free Dox or MMSN@Dox at a Dox concentration of 1 μg/mL. At predetermined time points (2 h, 4 h), the cells were washed, harvested, and resuspended in PBS to form single-cell suspensions. FCM analysis was subsequently performed to evaluate cellular uptake.

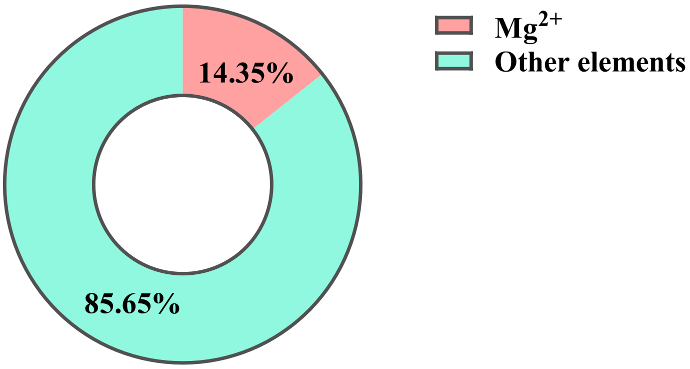

Figure S1. The Mg^{2+} content in MMSN measured by ICP-OES.

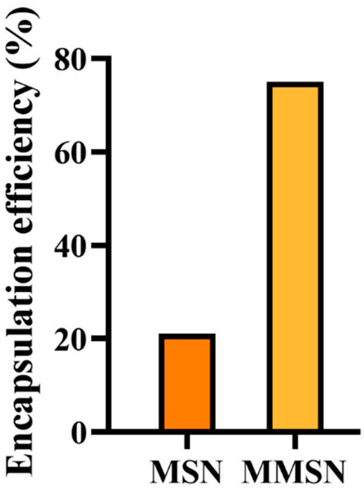

Figure S2. Encapsulation efficiency of MSN and MMSN.

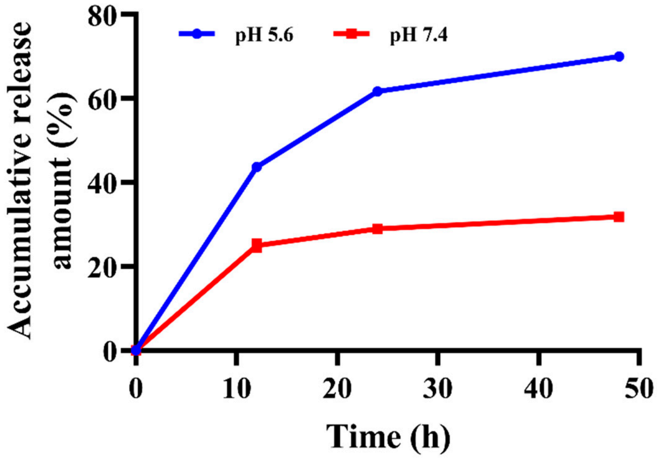

Figure S3. The cumulative release amount of Mg^{2+} by MMSN in different buffer solutions.

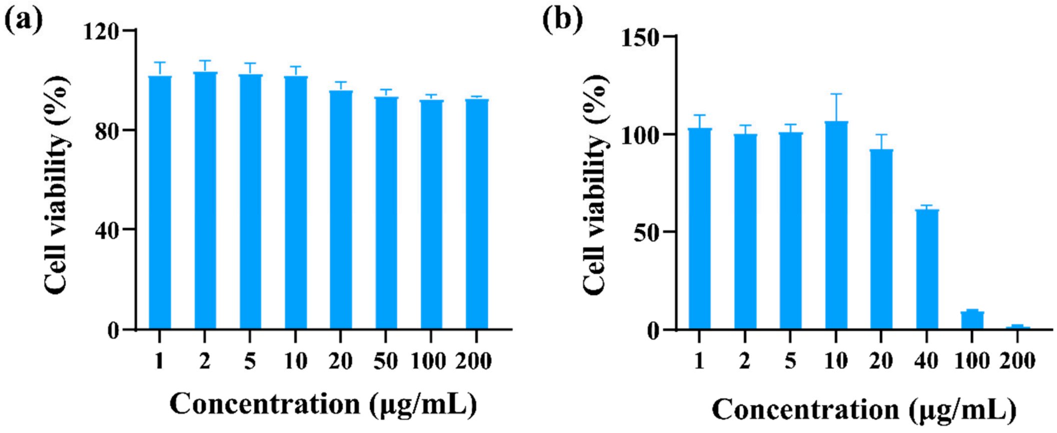

Figure S4. Cytotoxicity of (a) MSN and (b) MMSN toward 4T1 cells at different concentrations.

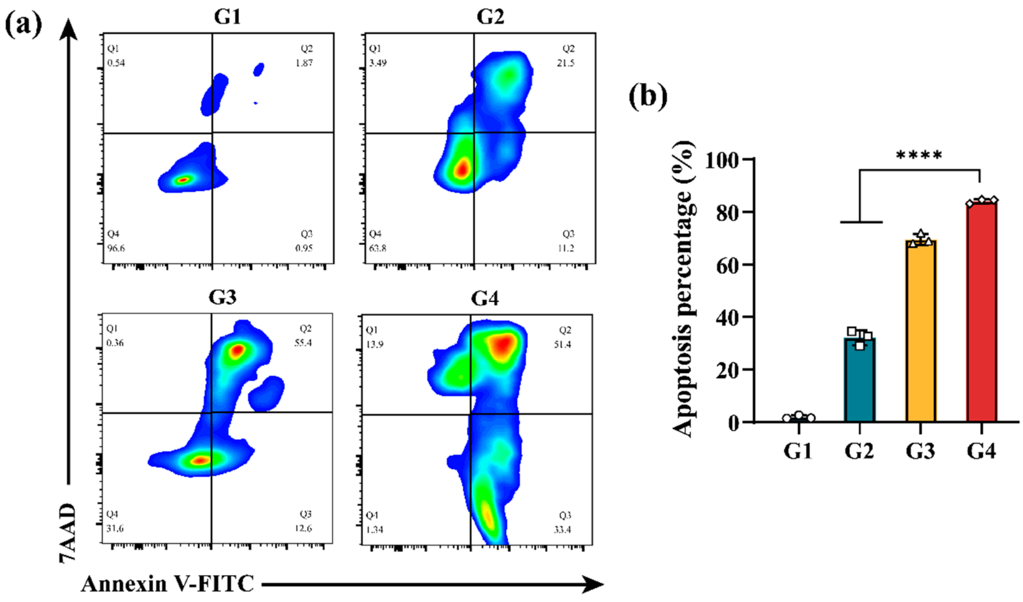

Figure S5. Apoptosis assay of 4T1 cells after being treated in different formulations for 24 h. (a) Representative FCM plots and (b) corresponding quantitative statistical results. Data are shown as Mean ± SD, n = 3, ****p <0.0001.

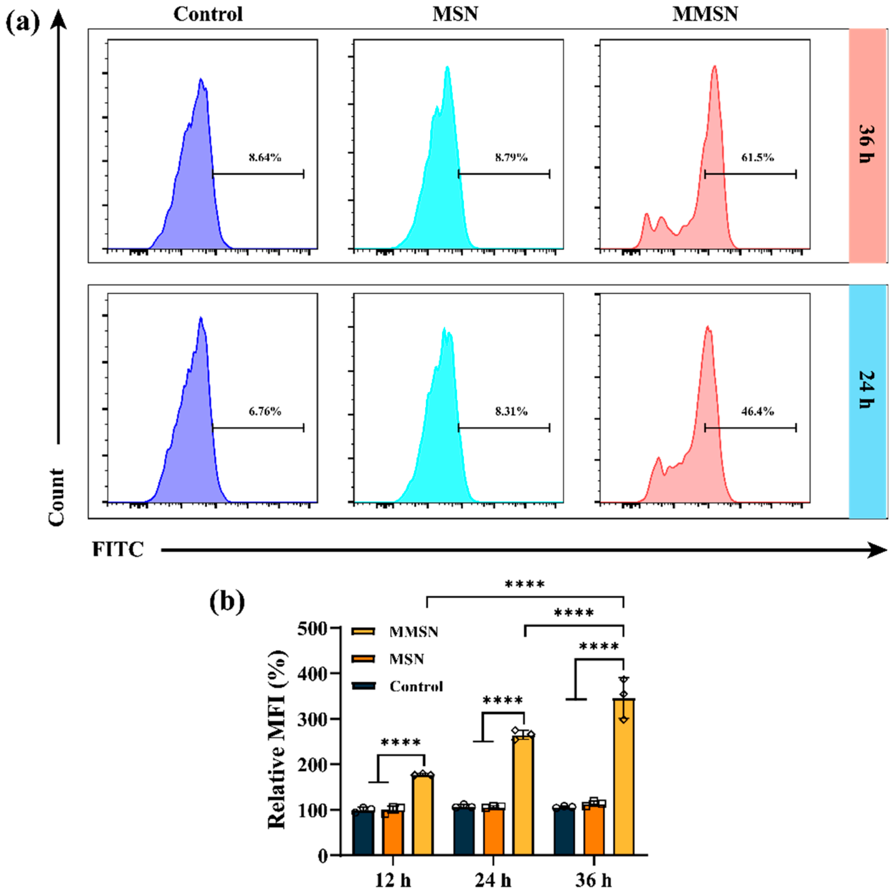

Figure S6. Measurement of ROS after different treatment durations. (a) FCM profiles of Intracellular ROS levels and (b) corresponding quantitative statistical results. Data are shown as Mean ± SD, n = 3, ****p <0.0001.

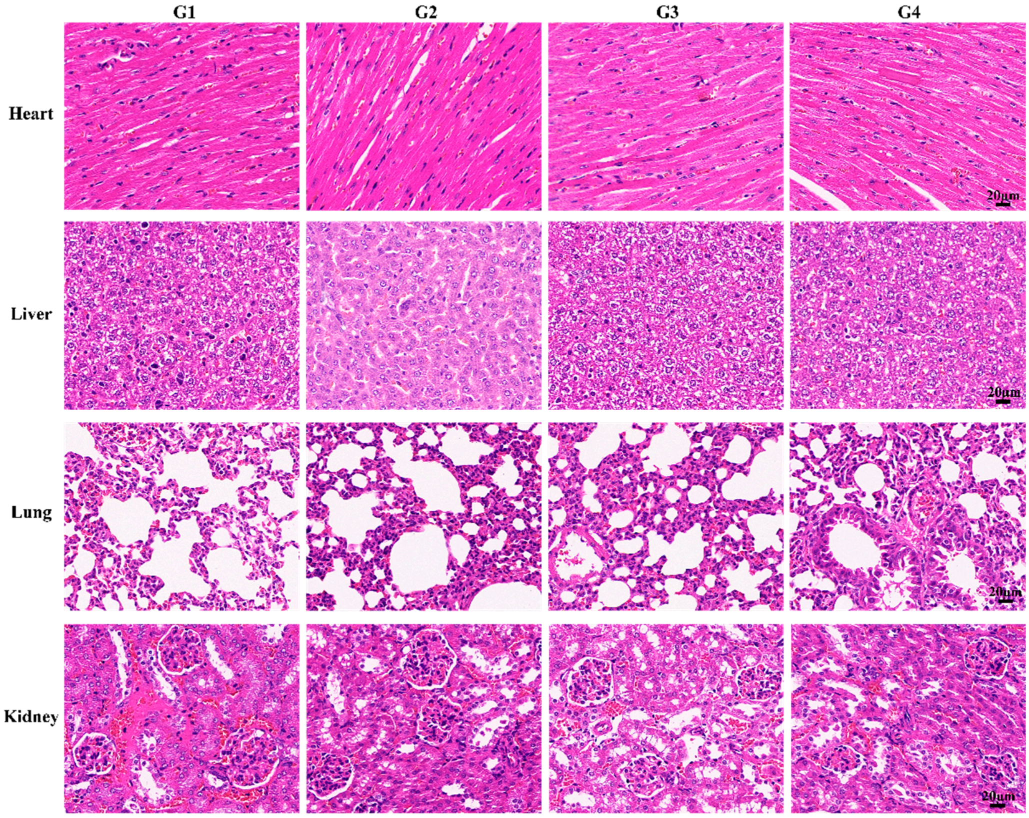

Figure S7. H&E staining images of main organs in each group.

References

44. Segall, M.; Lindan, P. J.; Probert, M. a.; Pickard, C. J.; Hasnip, P. J.; Clark, S.; Payne, M. J. J. o. p. c. m. First-principles simulation: ideas, illustrations and the CASTEPcode. *J. Phys.: Condens. Matter* **2002**, *14*, 2717-2744. http://doi.org/10.1088/0953-8984/14/11/301

45. Perdew, J. P.; Burke, K.; Ernzerhof, M. J. P. r. l. Generalized gradient approximation made simple. *Phys. Rev. Lett.* **1996**, *77*, 3865-3868. http://doi.org/10.1103/PhysRevLett.77.3865

46. Grimme, S.; Antony, J.; Ehrlich, S.; Krieg, H. A consistent and accurate ab initio parametrization of density functional dispersion correction (DFT-D) for the 94 elements H-Pu. *J. Chem. Phys.* **2010**, *132*, 154104-154122. http://doi.org/10.1063/1.3382344
